# Introducing GalaxyR: an easy-to-use R implementation of the Galaxy API

**DOI:** 10.1186/s12859-026-06591-0

**Published:** 2026-08-10

**Authors:** Julian Frey, Zoe Schindler, Muhammad Ali, Joshua Braun-Wimmer, Kilian Gerberding, Katja Kröner, Elena Larysch, Daniel Lusk, Janusch Vanja-Jehle, Yannik Wardius, Maximilian Weidenfeller, Teja Kattenborn, Thomas Seifert

**Affiliations:** 1 Chair of Forest Growth and Dendroecology, Freiburg University, 79106 Freiburg, Germany; 2 Chair of Sensor-based Geoinformatics, Freiburg University, 79106 Freiburg, Germany

**Keywords:** Software, Cloud computing, R programming language, Application programming interface, Galaxy, Workflow management

## Abstract

**Background:**

Standardisation, accessibility, and reproducibility remain persistent challenges in data‑intensive scientific research. The Galaxy platform addresses these issues by providing a web-based environment for executing, sharing, and publishing computational workflows, yet programmatic access has been largely centred on the Python ecosystem through BioBlend.

**Methods:**

Here, we introduce GalaxyR, a native R package that provides a comprehensive and structured interface to the Galaxy application programming interface. GalaxyR enables users to manage histories, upload and retrieve data, execute tools and workflows, monitor jobs, and inspect results directly from within the R environment. By integrating Galaxy’s scalable computational infrastructure with R’s widely adopted data analysis ecosystem, GalaxyR facilitates automated, reproducible, and resource‑efficient workflows without requiring local high‑performance computing resources. The package supports both HTTPS‑ and FTP‑based data transfer, robust history and dataset management, and programmatic workflow orchestration across Galaxy instances.

**Results:**

An application example demonstrates the use of GalaxyR for large-scale processing of drone-based laser scanning data, where computationally intensive tree‑level segmentation was delegated to the Galaxy infrastructure, while workflow control and preprocessing were handled in R within a few lines of code.

**Conclusions:**

GalaxyR thus bridges a critical gap for R users, significantly expanding access to Galaxy‑based analyses and enabling scalable, reproducible research across bioinformatics, ecology, remote sensing, and related data-driven disciplines.

## Background

Standardisation, accessibility, interoperability, and reusability of analytical tools remain central challenges in the life sciences and related data-driven research fields [1–3]. Addressing these challenges is essential for ensuring reproducibility, transparency, and long-term usability of computational analyses [2, 3]. The Galaxy platform represents one of the most comprehensive and successful efforts to tackle these issues by providing a unified platform for the execution, sharing, and publication of scientific workflows [4]. Originally established as a resource for biomedical research, Galaxy has since evolved into a general-purpose computational workbench widely used across biological, medical, ecological, remote sensing, and other earth system sciences [4, 5].

Galaxy is best known for its user-friendly, web-based interface that enables researchers to perform complex analyses without requiring programming expertise [6]. Beyond this no-code usage model, the platform provides access to substantial computational and storage resources, along with a rich ecosystem of community-maintained research data management and analysis tools and workflows. These capabilities support standardised and repeatable analyses, facilitate data and method sharing and publication, and promote reproducible research practices [4]. At the same time, Galaxy has been designed with automation in mind and exposes nearly all of its functionality through a well-documented representational state transfer (REST) application programming interface (API) [7, 8]. The REST API is implemented as an OpenAPI v3 [9], which ensures a standardised API design, automatic documentation generation, and code generation for multiple programming languages.

Direct interaction with a REST API, however, can be challenging for many scientists, particularly those whose primary expertise lies outside software engineering [8]. As a result, high-level software libraries that abstract the underlying API and integrate Galaxy functionality into familiar programming environments are of substantial value to the research community. Such abstractions are well established in the Python ecosystem [10] through the BioBlend package [8], and in further programming languages like Java, JavaScript or PHP, but not for the R programming environment [11]. The RGalaxy package [12] provides functionality to integrate R functions into Galaxy tools, but allows no direct interaction with the API. The R GalaxyConnector [13] allows uploading and downloading files from R to a Galaxy instance, but additional functionality, such as workflow and tool invocations, is absent. The lack of integration between Galaxy and R is notable given the widespread use of R across many scientific domains in which Galaxy plays an important role, including statistics-driven analyses, ecological modelling, and data-intensive life sciences [14, 15].

To address this limitation, we developed GalaxyR, a native R package that provides a structured and well-documented interface to the Galaxy API. GalaxyR enables users to programmatically upload data, create and manage histories, invoke tools and workflows, monitor execution, and retrieve results and metadata directly from within R. By integrating the Galaxy functionality into the R environment, the package allows researchers to automate complex analytical workflows while continuing to work within a familiar and widely adopted scientific programming framework. Importantly, because analyses can be executed on public Galaxy servers, GalaxyR enables users to overcome the need for dedicated local computing infrastructure, making large-scale or computationally intensive analyses accessible even to users without specialised hardware. This includes workflows that rely on substantial memory, parallel computation, or specialised accelerators, such as graphics processing units, for artificial intelligence models, thereby lowering technical barriers and broadening access to advanced analytical methods. Institutions might also choose to set up their own Galaxy instances to manage their tools, computational resources and load.

## Implementation of the GalaxyR package

The GalaxyR package is designed to support reproducible, scriptable, and scalable computational workflows for data-intensive scientific analyses, particularly in bioinformatics [5], geospatial data sciences [16–18] and related fields.

**Fig. 1:**
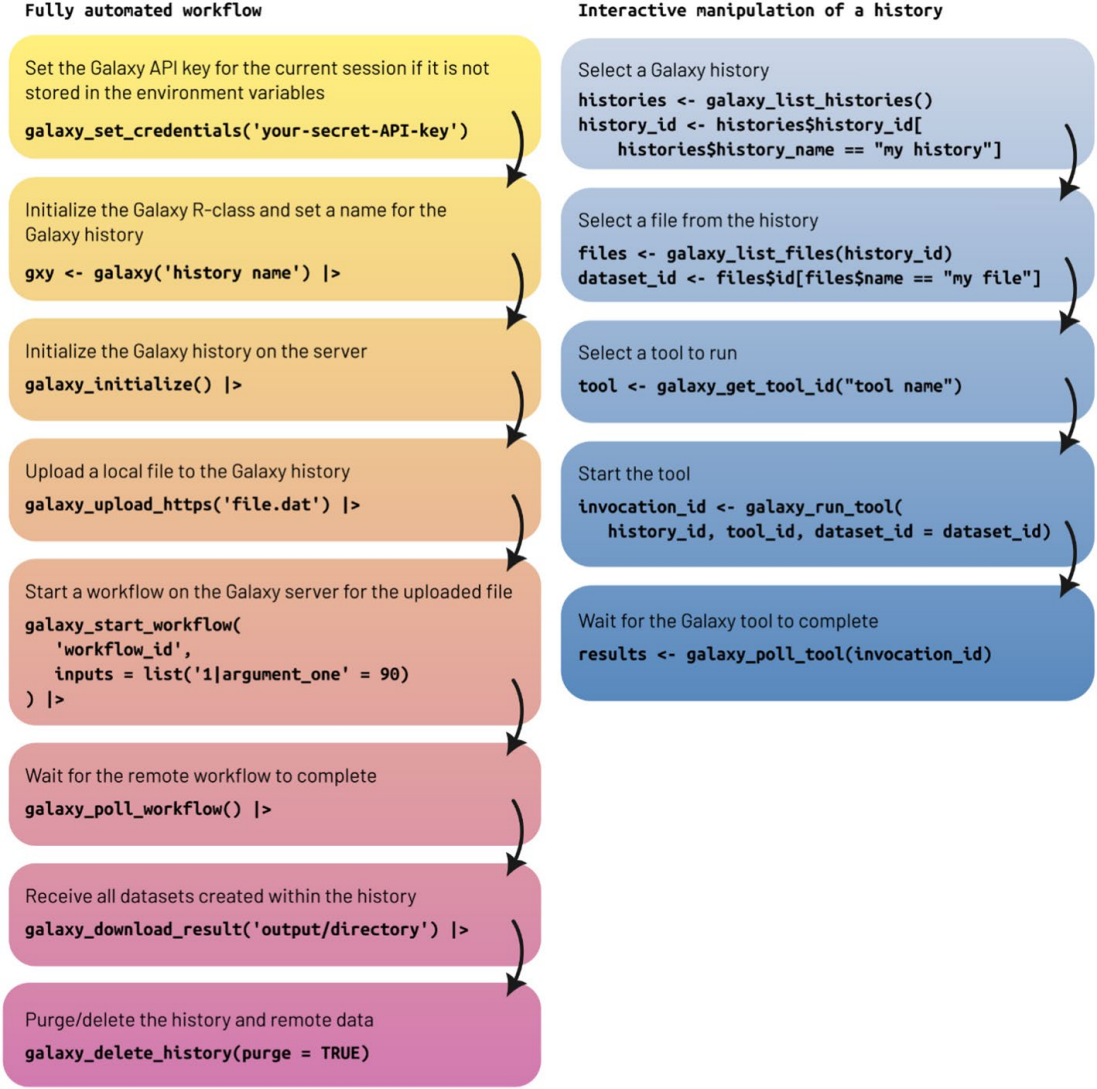
GalaxyR example command flow from initialisation (yellow) to results download (pink), or from inspection of a history (light blue) to the invocation of a tool and the inspection of the results (dark blue) from an existing history. The code shows the original R commands from the GalaxyR package

GalaxyR implements API-key-based authentication and centralised configuration of Galaxy connection parameters via environment variables, allowing seamless interaction with a specified Galaxy instance. It supports dynamic resolution of the Galaxy base URL and integrates credential handling for both HTTPS- and FTP-based data transfers. These design choices make GalaxyR suitable for use in interactive sessions as well as automated analysis pipelines (Fig. 1).

GalaxyR enables workflow invocations with just a few lines of code. The main functionality is implemented as an S4 class that holds all relevant information for executing the next steps and can therefore be piped through the workflow in a comfortable way (Fig. 1).

GalaxyR provides comprehensive support for Galaxy history management. Users can create new histories, list existing histories, and compute disk usage for individual histories in a manner that is robust to differences across Galaxy server versions. Disk usage is reported in both raw byte counts and human-readable formats, facilitating resource monitoring in large-scale analyses.

Data upload is supported through multiple mechanisms. Local files can be uploaded via HTTPS using Galaxy’s built-in upload tools, with optional polling until the uploaded datasets are fully processed and ready for downstream use. For large files or environments where HTTPS uploads are restricted, FTP-based uploads are also supported. These capabilities allow GalaxyR to accommodate a wide range of data sizes and deployment scenarios.

Single tools and workflows installed on the Galaxy instance can be listed, filtered, and queried for detailed metadata, including input and output specifications. Tool and workflow identifiers can be resolved by name, facilitating reproducible tool selection across Galaxy instances. Tools and workflows can then be executed with fully specified input parameter sets, closely mirroring Galaxy’s internal JSON-based execution model. GalaxyR includes functionality to monitor the execution by polling Galaxy jobs until they reach a terminal state, thereby ensuring reliable synchronisation between R scripts and Galaxy’s asynchronous execution model. Upon completion, GalaxyR retrieves the identifiers of output datasets generated by the execution, enabling seamless chaining of analyses.

GalaxyR also provides functionality for dataset inspection and management. For one or multiple Galaxy datasets, the package can retrieve metadata including dataset name, file type, size in bytes, human-readable size, processing state, and deletion status. This information is returned in tabular form, making it straightforward to integrate into downstream analyses or reporting workflows. Result datasets produced by tools or workflows can be downloaded directly to local files, enabling further processing outside of Galaxy. Finally, the package includes utilities for dataset cleanup, allowing individual datasets or collections of datasets to be deleted programmatically, with optional permanent purging to free disk space. Requests are paced to avoid overloading the Galaxy server, reflecting an emphasis on robustness and responsible API usage.

## Application example

**Fig. 2:**
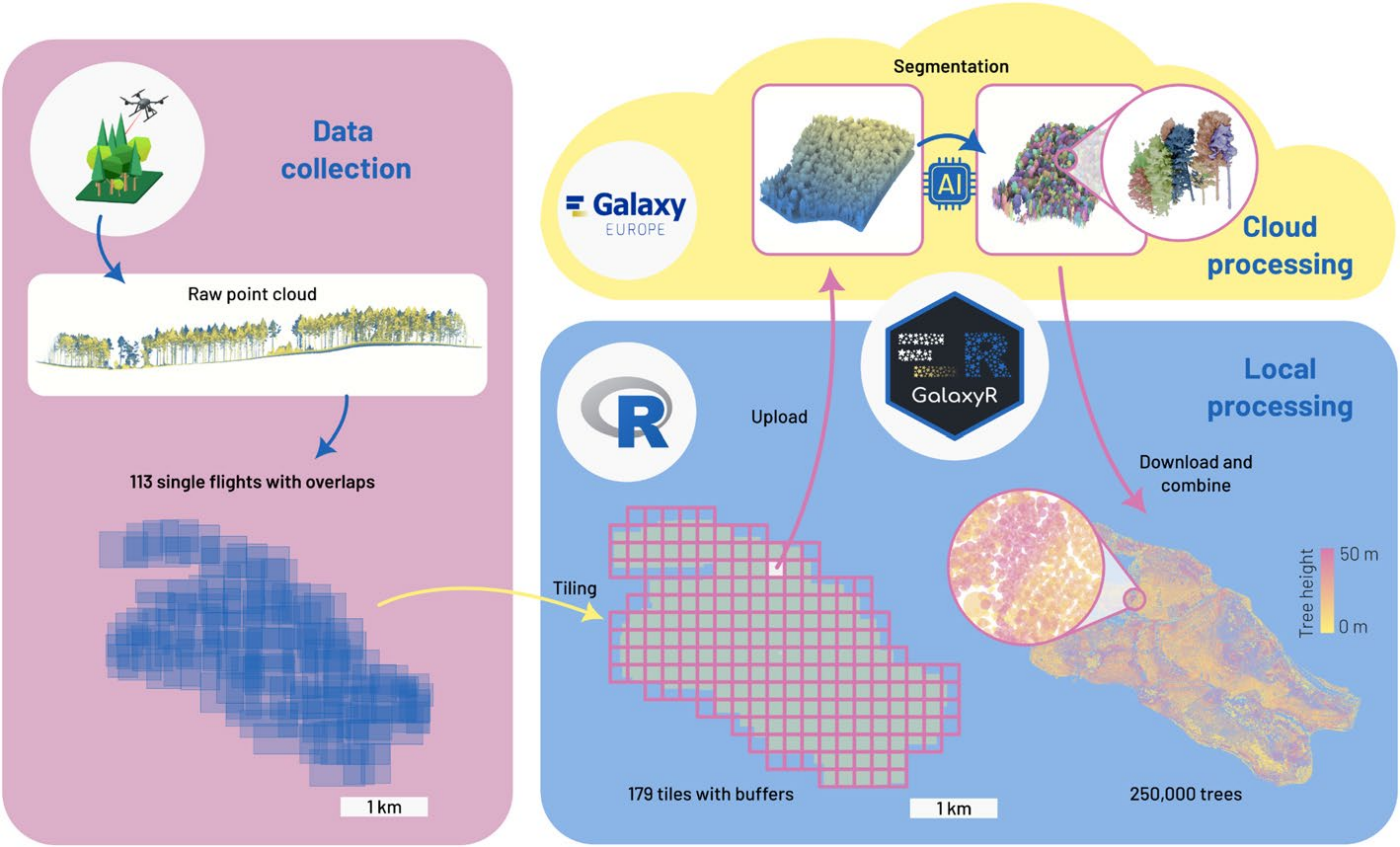
Workflow of the case study. A LiDAR dataset has been collected in multiple flights utilising a multirotor UAV on a 600 ha forested landscape (rose box). In the R environment, flights have been retiled to minimise overlap (blue box) and automatically uploaded to the Galaxy Europe platform using GalaxyR for AI-based instance segmentation of trees (yellow cloud). Afterwards, the segmented point clouds have been downloaded and analysed locally to create a single tree map of the whole area (blue box)

Forests provide essential ecosystem services—carbon sequestration, biodiversity support, water regulation, and timber production—yet their complex three‑dimensional structure makes reliable, large‑scale monitoring a persistent challenge [19]. The structural mapping of forests by modern remote sensing techniques such as Light Detection and Ranging (LiDAR) has made great progress in recent decades, but processing such big datasets still proves to be difficult [20].

In spring 2025, we conducted 113 flights using a multirotor uncrewed aerial vehicle UAV (DJI Matrice 350, DJI, China) equipped with a LiDAR sensor (DJI L2, DJI, China) over a forested area in the central‑western part of Germany. The objective was to obtain a high‑resolution representation of forest structure, with a particular focus on single‑tree attributes such as tree height. This close‑range remote sensing approach produced centimetre‑accurate, dense three‑dimensional point clouds representing individual LiDAR returns within the forest canopy and understory (Fig. 2).

The resulting dataset covered more than 600 hectares of mountainous forest and comprised approximately 4.7 billion points, corresponding to more than 460 gigabytes of compressed data after initial preprocessing. Extracting single‑tree parameters from data of this scale required the application of an instance segmentation model specifically designed for tree‑level analysis [21], which exceeded the available local computing resources.

Within the R programming environment, the lidR package provides a high‑performance tiling and processing framework for large point cloud datasets, including support for overlapping tiles [22]. We used lidR to reorganise our data into 140 tiles with an edge length of 200 m, replacing the original structure of one file per flight with overlapping coverage at tile boundaries. An additional buffer of 10 m was added around each tile to prevent edge artefacts during processing (Fig. 2). The computationally intensive single‑tree instance segmentation was then executed on the European Galaxy servers, using the GalaxyR package directly from within the lidR tiling workflow. For each of the 140 tiles, we fully automatically created the history and started the workflow.

After downloading the segmentation results from the Galaxy instance, all remote data were deleted, and trees outside the tile boundaries were removed automatically. Using this approach, we were able to process the complete dataset and segment more than 250,000 individual trees within a total runtime of 72 h. The R code necessary for this analysis is just about 40 lines long, underscoring how efficiently tasks can be automated using GalaxyR (Appendix 1).

This application example illustrates how combining R and Galaxy enables efficient, scalable analysis of large, complex datasets that exceed local computational capabilities. By using R as an orchestration and preprocessing environment and delegating computationally intensive tasks to Galaxy, researchers can seamlessly integrate local data handling with remote, high-performance execution. The tight coupling provided by GalaxyR allows workflows to be defined, parameterised, and monitored directly from R, while Galaxy transparently manages data transfer, scheduling, and execution on suitable infrastructure. This division of labour leverages the strengths of both environments: R for flexible data manipulation and workflow control, and Galaxy for robust, reproducible execution at scale.

Since this application example requires a very large dataset and high computational demands, we have also added a lightweight example directly within the package and in Appendix 2.

## Discussion

While GalaxyR provides a broad set of functions for interacting with Galaxy histories, tools, workflows, and the retrieval of structured provenance records (RO-Crate) [23], several capabilities described below are not yet supported. As a result, users who require these functionalities must currently rely on Galaxy’s web interface or external tools.

Support for more complex data structures is limited. Although workflows and tools that operate internally on grouped or paired datasets can be executed, GalaxyR does not yet provide dedicated functions to create, inspect, or manipulate such dataset groupings directly from R [7]. This limits the ability to programmatically manage analyses that rely heavily on structured collections of input data.

In addition, GalaxyR primarily focuses on file‑based data stored in Galaxy histories. Direct interaction with remote data sources, such as externally hosted repositories or federated storage systems accessible through Galaxy, is not yet implemented. As large‑scale and distributed data access becomes increasingly important, extending GalaxyR to better support remote data integration represents a natural direction for future development. Nevertheless, users can currently set external data sources, such as S3 buckets, as their default storage in Galaxy, which will also be used by GalaxyR.

Future work on GalaxyR will therefore focus on expanding support for improving the handling of complex dataset structures and enabling more seamless access to remote data resources. We aim to actively maintain and further improve the package, and we also invite the user community to contribute to the codebase.

Note that the provenance records created by Galaxy (RO-crates) only contain the part of the computations that have been executed within the Galaxy environment. Any further data manipulations and analyses done in the local R session will not be tracked. Nevertheless, users can use markdown tools such as Quarto or Jupyter to record the processing steps from their local R session. Additionally, RStudio can be run directly in Galaxy as an interactive tool within the same history, resulting in a full provenance record if the R history is saved as a markdown document [24]. GalaxyR is available in the RStudio interactive tool on Galaxy via the CRAN repository.

## Conclusion

In summary, GalaxyR provides an end-to-end R interface to the Galaxy API, covering history management, data upload, tool execution, workflow orchestration, result retrieval, and resource monitoring. Its design emphasises reproducibility, automation, and compatibility across Galaxy instances, making it a suitable foundation for integrating Galaxy-based analyses into scientific R workflows and computational pipelines. This significantly strengthens the ability of the R users’ community to utilise the platform for various applications, from simple tool usage for tools not available on R to complex automation tasks or big data analyses with high computational demands. Additionally, it offers a flexible interface for Galaxy users to connect their analyses to downstream R applications and tools.

## Availability and requirements

Project name: GalaxyR.

Project home page: https://github.com/JulFrey/GalaxyR.

Operating system(s): Platform independent.

Programming language: R.

Other requirements: R4.2 or higher.

License: GNU GPL.

Any restrictions to use by non-academics: None.

GalaxyR is published on the CRAN R package archive. Additionally, a Conda package (https://anaconda.org/conda-forge/r-galaxyr*)* and a Docker container https://quay.io/repository/biocontainers/r-galaxyr are available.

## Data Availability

All code is published. The LiDAR data sets exceed the limits of public repositories and are therefore available upon request.

## Abbreviations

API: Application programming interface
REST: Representational state transfer
FAIR: Findable, accessible, interoperable, reusable
UAV: Unmanned/uncrewed aerial vehicle
LiDAR: Light detection and ranging
TLS: Terrestrial laser scanning
HPC: High-performance computing
GPU: Graphics processing unit
